# Impact of the level of complexity in self-sorting: Fabrication of a supramolecular scalene triangle

**DOI:** 10.3762/bjoc.7.183

**Published:** 2011-11-22

**Authors:** Kingsuk Mahata, Michael Schmittel

**Affiliations:** 1 Center of Micro- and Nanochemistry and Engineering, Organische Chemie I, Universität Siegen, Adolf-Reichwein-Straße, D-57068 Siegen, Germany

**Keywords:** copper, metallosupramolecular chemistry, phenanthroline, self-assembly, self-sorting

## Abstract

The impact of the level of complexity in self-sorting was elaborated through the fabrication of various scalene triangles. It turned out that the self-sorting system with a higher level of complexity was far superior to less complex sorting algorithms.

## Introduction

**Scheme 1:**
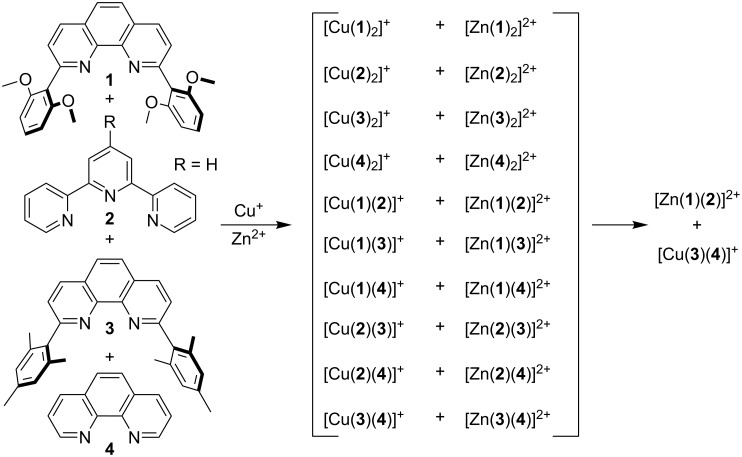
Self-sorting in a six-component library [8].

Self-assembly guided by self-sorting algorithms has received considerable attention over the past two decades as such protocols pave the way for intricate supramolecular assemblies [1–19]. However, despite its wide use, the definition of self-sorting remains vague with no precise guidelines provided in the literature. It has been widely reported that self-sorting operates when the numerical outcome of a chemical system is lower than the plausible number of potential aggregates (assemblies) estimated on the basis of statistical, chemical, and geometrical arguments [20–21]. This definition has drawbacks, and levelling effects [22] have been observed among various self-sorting systems. To numerically grasp the difference between different self-sorting processes, we defined the degree of self-sorting *M* as *M* = *P*/*P*_0_ with *P* representing the number of possibilities and *P*_0_ representing the number of experimentally observed aggregates in the mixture [8]. For example, the degree of the self-sorting process realised with ligands **1**–**4** in the presence of both Cu^+^ and Zn^2+^, as described in Scheme 1, is *M* = 10, as only two complexes formed out of twenty possible ones [8].

**Scheme 2:**
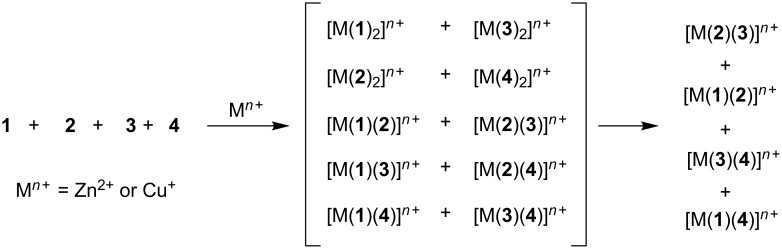
Self-sorting in a five-component library. We used **2** in a slightly different form with Zn^2+^ (R = 4-C_6_H_4_I) and Cu^+^ (R = H).

In contrast, when ligands **1**–**4** were treated with either Cu^+^ or Zn^2+^, the observed experimental outcome was four complexes (Supporting Information File 1, Figures S1 and S2), which has to be evaluated in light of the ten possible products (Scheme 2). Thus, for this process *M* = 2.5. Is this difference in *M* of any relevance, for example in the fabrication of intricate entities, or not? Herein, we investigate the utility of both self-sorting algorithms, from Scheme 1 and Scheme 2, for the fabrication of supramolecular scalene triangles. Importantly, we are able to demonstrate that the clean formation of a scalene triangle is only possible with the algorithm exhibiting the higher degree of self-sorting.

**Figure 1:**
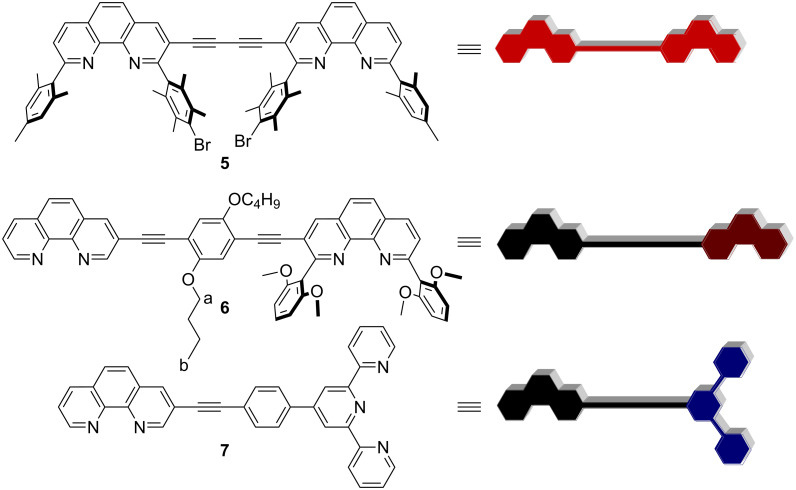
Ligands used in the present study.

Among the triangular assemblies, the scalene triangle is found to be the most difficult to fabricate. Hence, it comes as no surprise that there is only one report on a scalene triangle so far [16]. To design a further scalene triangle we modified a design already probed in the preparation of a geometrically isosceles triangle [15], and we implemented the coordination motifs of **1**–**4** into the three different multitopic ligands **5**–**7** [15,23], integrating twice the [Cu(**3**)(**4**)]^+^ [24–26] and once the [Zn(**1**)(**2**)]^2+^ motif [8,16] (Figure 1). The coordination behaviour of molecular component **3** was integrated into **5**, the latter being synthesised in a Sonogashira homocoupling reaction [27]. The information stored in **1** and **4** was instated in the unsymmetrical bisphenanthroline **6**, readily accessible by stepwise Sonogashira cross-coupling reactions [15]. A known procedure was followed to prepare the terpyridine–phenanthroline hybrid **7** [8]. The lengths of the ligands were chosen in such a way that they provide the geometrically different sides of a scalene triangle.

## Results and Discussion

**Scheme 3:**
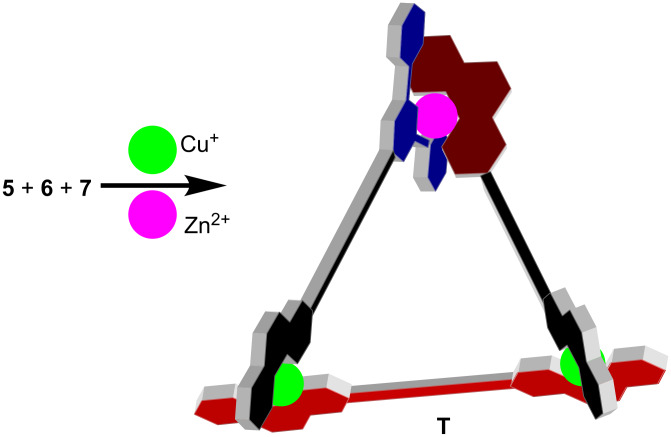
Synthesis of the triangular assembly **T** (only *syn* shown).

**Figure 2:**
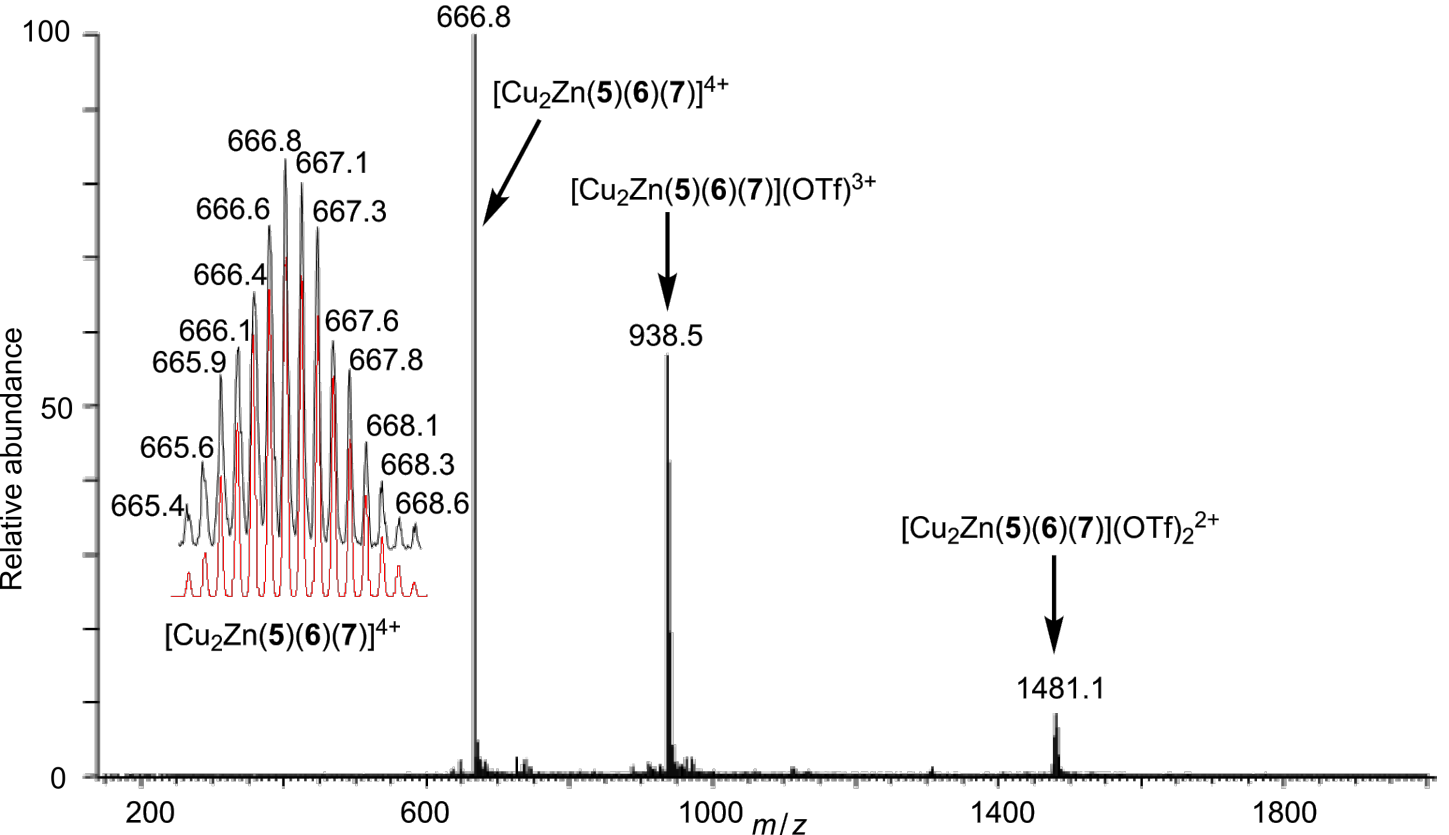
ESI-MS of triangle **T** in acetonitrile along with the isotopically resolved peak at 666.8 (black: Experimental; red: Calculated for [Cu_2_Zn(**5**)(**6**)(**7**)]^4+^). See also Figure S5 in Supporting Information File 1.

We tested both self-sorting algorithms as described earlier (Scheme 1 and Scheme 2). In a first round of experiments, we combined the ligands **5**, **6**, and **7** in equimolar ratio and made them react with one equivalent of Zn^2+^ and two equivalents of Cu^+^ in acetonitrile at 60 °C for 3 h. At the end, a clear red solution was furnished, which was characterised as received, by means of mass spectrometry, ^1^H NMR, diffusion-ordered spectroscopy (DOSY), differential pulse voltammetry (DPV) and elemental analysis. The electrospray ionisation mass spectrum (ESI-MS) of the reaction mixture suggests clean formation of the triangular species **T** = [Cu_2_Zn(**5**)(**6**)(**7**)](OTf)_2_(PF_6_)_2_ (Scheme 3). In the accessible spectral region of *m*/*z* = 150–2000 only three intense peaks were observed, all of them corresponding to triangle **T** (Figure 2). The most abundant peak at *m*/*z* = 666.8 can be assigned to [Cu_2_Zn(**5**)(**6**)(**7**)]^4+^, whereas the triply charged one at *m*/*z* = 938.5 is attributed to [Cu_2_Zn(**5**)(**6**)(**7**)](OTf)^3+^, and the doubly charged one at *m*/*z* = 1481.1 to [Cu_2_Zn(**5**)(**6**)(**7**)](OTf)_2_^2+^. All peaks were isotopically resolved, showing full agreement with the theoretically expected isotopic distribution.

**Figure 3:**
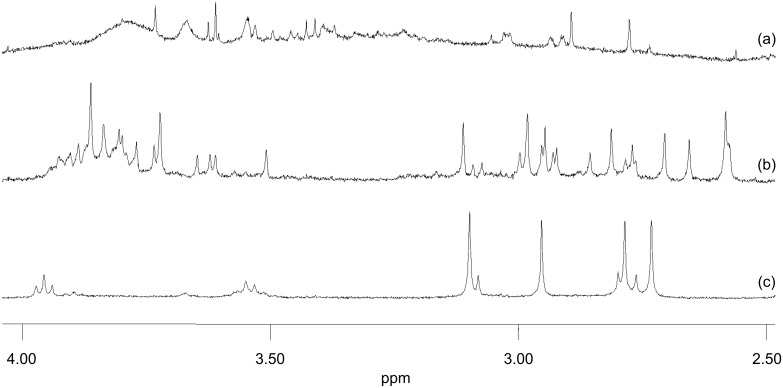
Partial ^1^H NMR (400 MHz, 298 K, CD_3_CN) spectra of an equimolar mixture of **5**, **6**, and **7** in the presence of (a) 3 equivalents of Cu^+^, (b) 3 equivalents of Zn^2+^ and (c) 3 equivalents of a metal-salt mixture (Cu^+^:Zn^2+^ = 2:1).

To corroborate the clean self-assembly process, we carefully examined the DOSY and ^1^H NMR of **T**. As in the ESI-MS, both sets of data unambiguously supported the presence of only one species, i.e., the DOSY spectrum showed only a single diffusion coefficient (Supporting Information File 1, Figure S4). Additional information was derived from the ^1^H NMR signals of the methoxy protons, as these appear in a diagnostic region. In **T**, up to eight singlets are expected for the four methoxy groups due to their constitutional differences and the occurrence of two diastereomers (*syn* and *anti*). Diastereomers form as a result of two stereogenic heteroleptic copper(I) complex motifs [Cu(**3**)(**4**)]^+^ in **T** [8,28–29]. The ^1^H NMR of the assembly indeed showed seven singlets (one peak is merged with the others) between 2.73–3.10 ppm (Figure 3c). From NMR integration the ratio of the diastereomers was found to be approximately 3:1. The finding of further characteristic ^1^H NMR shifts for protons of **6** (H–a and H–b) additionally supports the formation of **T** as a mixture of two diastereomers. Four triplets (two for each diastereomer) were observed for protons H–a between 3.53–3.97 ppm, and the same number of triplets was seen for H–b in the region of 0.57–1.00 ppm (Supporting Information File 1). Elemental analysis of the assembly also confirmed the exclusive formation of **T**.

**Figure 4:**
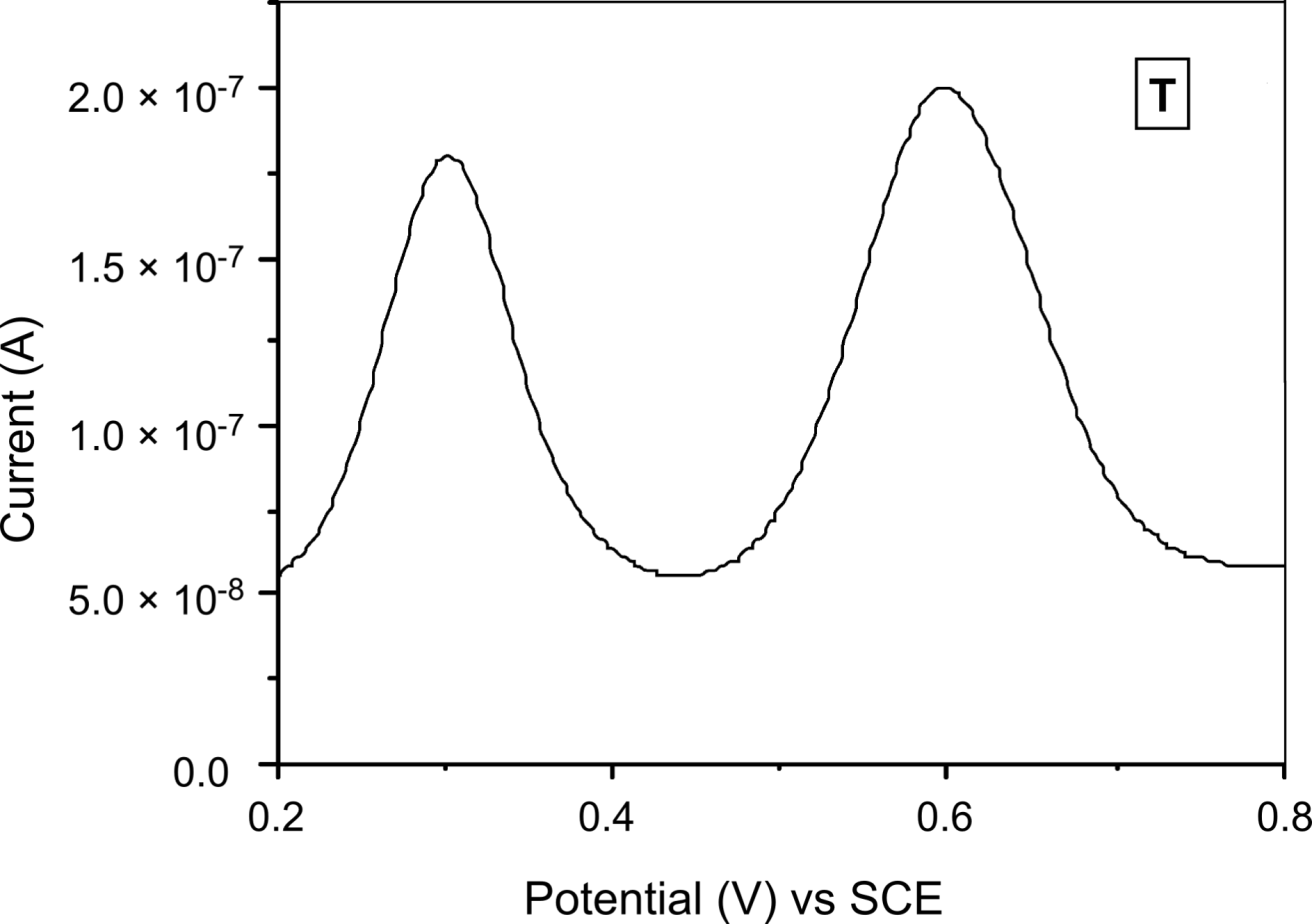
Differential pulse voltammogram of **T** in acetonitrile (0.1 M *n*-Bu_4_NPF_6_ as electrolyte, Ag wire as a quasi-reference electrode, 1,1'-dimethylferrocene as internal standard, scan rate = 20 mV s^−1^ and a pulse height of 2 mV).

We further evaluated the structure using DPV, because this analytical method provides valuable information about redox active units (here Cu^+^). It is well known that copper(I) shows distinct oxidation potentials in different complex environments. Thus, the DPV measurements should allow us to analyse the number and ligand sphere of copper(I) centres in **T**. In the mononuclear complex [Cu(**1**)(**4**)](PF_6_) the copper(I) oxidation is observed at +0.29 V_SCE_, whereas for [Cu(**3**)(**4**)](PF_6_) and [Cu(**1**)(**2**)](PF_6_) the oxidation is placed at +0.44 V_SCE_ and −0.21 V_SCE_, respectively [8]. In **T**, only one type of copper(I) complex is present. As two values were expected for the two diastereomers, the broad peak was deconvoluted for two copper(I) oxidation waves (Figure 4) resulting in two values at +0.59 and +0.64 V_SCE_. The values agree with those reported for a similar copper(I) complex (+0.61 and +0.67 V_SCE_) in a recently reported supramolecular trapezoid [8]. The population of the two diastereomers as determined from the deconvoluted DPV spectrum (Supporting Information File 1) was roughly 3:1, in full agreement with the ratio derived from ^1^H NMR results.

We then focused on the self-sorting protocol mentioned in Scheme 2. We reacted three equivalents of copper(I) ions with an equimolar mixture of all three ligands **5**–**7**. After 3 h, at similar conditions as for **T**, a dark red solution was afforded, which was characterised by ^1^H NMR without any further purification. The ^1^H NMR spectrum was found to be broad (Figure 3a). The broadening of the signals is partly due to the presence of a phenanthroline–Cu^+^–terpyridine complex. Due to the tetrahedral coordination behaviour of Cu^+^, one pyridine nitrogen atom of the terpyridine unit is left uncoordinated [30], and thus it undergoes rapid exchange leading to broad NMR signals. The experiment was also carried out in the presence of three equivalents of Zn^2+^. A clear yellow solution was produced after exposure to similar reaction conditions. Unlike the other experiment with Cu^+^, only sharp signals were observed in the ^1^H NMR (Figure 3b), but the many signals in the region 2.5–4.0 ppm suggest formation of several species. Thus, a comparison among the NMR spectra nicely demonstrated that the self-assembly process was only clean in the case of the mixed-metal scenario, whereas the situation turned out to be complicated in both homometallic cases.

The observations can be rationalised in the following way. In the homometallic cases selectivity was less, because the ligands may organise into ≥2 competing triangular arrays with different connectivities (constitutions). In the all-copper situation, the linkage between **5** and **6** is only possible by [Cu(**3^5^**)(**4^6^**)]^+^ coordination [31]. The triangle is completed through a bridging with **7**. However, the connectivity of **7** is not defined. The ligand may arrange itself in either of the two possible ways, [Cu(**3^5^**)(**4^7^**)]^+^ and [Cu(**1^6^**)(**2^7^**)]^+^ or [Cu(**2^7^**)(**3^5^**)]^+^ and [Cu(**1^6^**)(**4^7^**)]^+^, as demonstrated in Scheme 2, resulting in the formation of two different triangular species. Hence, the number of constitutional isomers increases in the case of the all-copper triangle. A related explanation may be given for the all-zinc triangle. The hindered phenanthroline of **6**, i.e., unit **1**, has four methoxy groups available for coordination in addition to its two bisimine nitrogens. Thus, unit **1** may either act as a strong bidentate, tridentate or tetradentate binding site for zinc(II) ions, and there is no large thermodynamic difference between a [Zn(**1^6^**)(**2^7^**)]^+^-type connection and a [Zn(**1^6^**)(**4^7^**)]^+^-type link. The outcomes are similar to those observed with copper(I) ions. On the other hand, in the mixed-metal scenario the terpyridine prefers to connect with terminus **1** as embedded in **6**, which is nicely illustrated from the self-sorting described in Scheme 1. Thus, the self-assembly process was constitutionally clean when self-sorting occurred along the algorithm with the higher level of complexity (Scheme 1). Due to the beauty of self-sorting, the five-component assembly (five different starting materials, mixed metal scenario) was flawless as compared to the four-component assembly (four different starting materials, homometallic cases).

**Figure 5:**
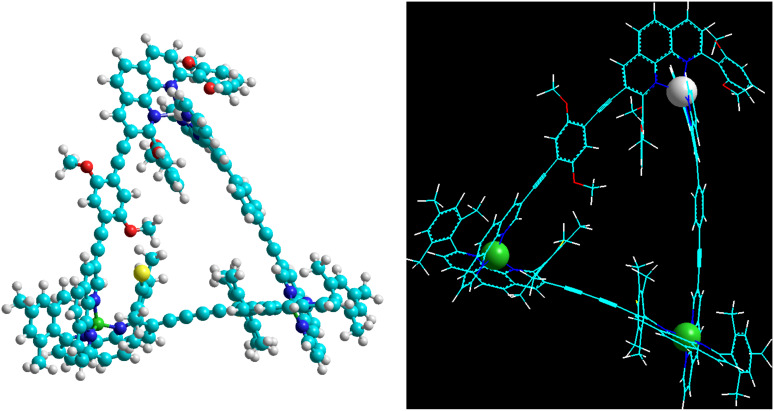
Two representations of the energy-minimised structure of the scalene triangle **T** (*anti*); copper(I) ions – green, zinc(II) ion – white.

As all attempts to obtain a crystal structure of **T** were unsuccessful, MM^+^ force-field computations and molecular dynamics on **T** (Hyperchem 7.52^®^, Hypercube, Inc.) provided some insight to their structure as scalene triangles. Taking the metal–metal distance as a measure, the three metal corners of **T** (*syn*) are separated by 1.27, 1.58 and 1.63 nm in the energy-minimised structure (Figure 5) and by 1.36, 1.58 and 1.63 nm in **T** (*anti*) (Supporting Information File 1), nicely illustrating the scalene triangle arrangement of **T**.

## Conclusion

We have been able to establish that our strategy to fabricate an isosceles triangle [15] can equally be applied to the preparation of a supramolecular scalene triangle and thus is tolerant to changes at the angles (of the vertices) and to variations of the lengths of the sides. Moreover, we have demonstrated with a study on homo- versus heterometallic scalene triangles that the level of complexity of self-sorting is important for the fabrication of intricate supramolecular assemblies [32–33].

## Experimental

### General

All commercial reagents were used without further purification. The solvents were dried with appropriate desiccants and distilled prior to use. NMR measurements were carried out on a Bruker Avance 400 MHz spectrometer with the deuterated solvent as the lock and residual solvent as the internal reference. Electrospray ionisation mass spectra (ESI-MS) were recorded on a Thermo-Quest LCQ Deca. Differential pulse voltammetry (DPV) was measured on a Parstat 2273 in dry acetonitrile. The melting point was measured on a Büchi SMP-20 and is uncorrected. The infrared spectrum was recorded on a Varian 1000 FT-IR instrument and the elemental analysis measurement was performed with a EA 3000 CHNS. Compound **5** [27], **6** [15], and **7** [8] were synthesised according to known procedures.

### Synthesis of scalene triangle T

**6** (1.49 mg, 1.65 μmol), **5** (1.76 mg, 1.65 μmol), **7** (0.85 mg, 1.65 μmol), Zn(OTf)_2_ (0.60 mg, 1.66 μmol) and [Cu(MeCN)_4_]PF_6_ (1.23 mg, 3.31 μmol) were heated under reflux in a mixture of dichloromethane (10 mL) and acetonitrile (25 mL) for 2 h. The solvents were evaporated under reduced pressure and the solid was characterised as such. Yield quantitative; mp >260 °C; IR (KBr) ν: 3448, 3068, 2953, 2931, 2869, 2362, 2209, 1617, 1602, 1589, 1549, 1499, 1475, 1427, 1406, 1383, 1277, 1255, 1223, 1159, 1111, 1030, 1019, 912, 843, 791, 767, 725, 639; ESI-MS *m*/*z* (%): 666.8 (100) [M − 2PF_6_, 2OTf]^4+^, 938.5 (70) [M − 2PF_6_, OTf]^3+^, 1481.1 (20) [M − 2PF_6_]^2+^; Anal. calcd for C_161_H_127_Br_2_Cu_2_F_18_N_13_O_12_P_2_S_2_Zn·3CH_2_Cl_2_: C, 56.10; H, 3.82; N, 5.19; S, 1.83; found: C, 56.36; H, 3.27; N, 5.35; S, 1.91.

## Supporting Information

File 1 ^1^H NMR spectra of self-sorting mixtures and of **T**. DOSY, ESI and DPV spectra of **T**.
