# Supplementary material for: Impact of the level of complexity in self-sorting: Fabrication of a supramolecular scalene triangle
Source: Beilstein J Org Chem. 2011 Nov 22;7:1555–61. doi: 10.3762/bjoc.7.183 (PMC3252858; doi:10.3762/bjoc.7.183)
Supplement: File 1 — 1H NMR spectra of self-sorting mixtures and of T. DOSY, ESI and DPV spectra of T. [file Beilstein_J_Org_Chem-07-1555-s001.pdf]

Supporting Information

for

Impact of the level of complexity in self-sorting: Fabrication of a supramolecular scalene triangle

Kingsuk Mahata and Michael Schmittel*

Address: Center of Micro- and Nanochemistry and Engineering, Organische Chemie I, Universität Siegen, Adolf-Reichwein-Straße, D-57068 Siegen, Germany.

Email: Michael Schmittel* - schmittel@chemie.uni-siegen.de

* Corresponding author

^{1}H NMR spectra of self-sorting mixtures and of T. DOSY, ESI and DPV spectra of T.

Table of contents

1 NMR spectra, DOSY S2

2 ESIMS spectra S5

3 DPV S6

4 Deconvolution S6

5 Computational results S8

NMR spectra

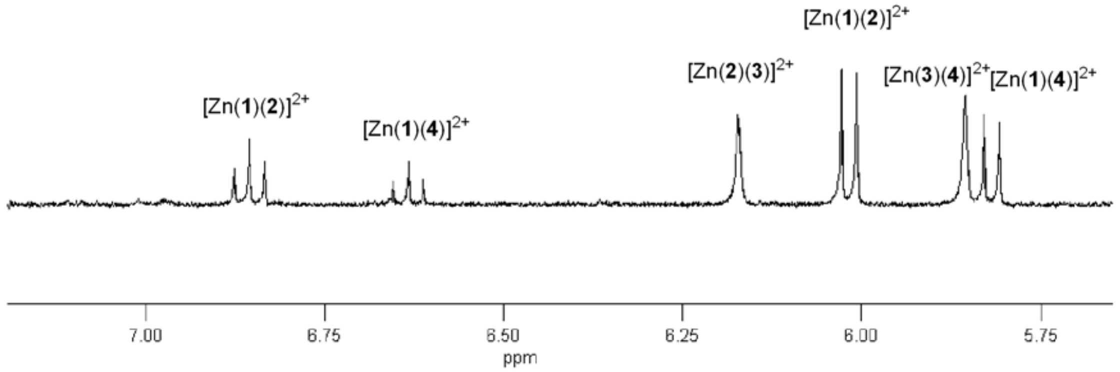

Figure S1: Partial ^{1}H NMR spectrum ($CD_3CN/CDCl_3$ = 3:1, 298 K) of an equimolar mixture of **1**, **2** (R = 4-C_6H_4I), **3** and **4** in the presence of two equivalents of Zn^{2+}.

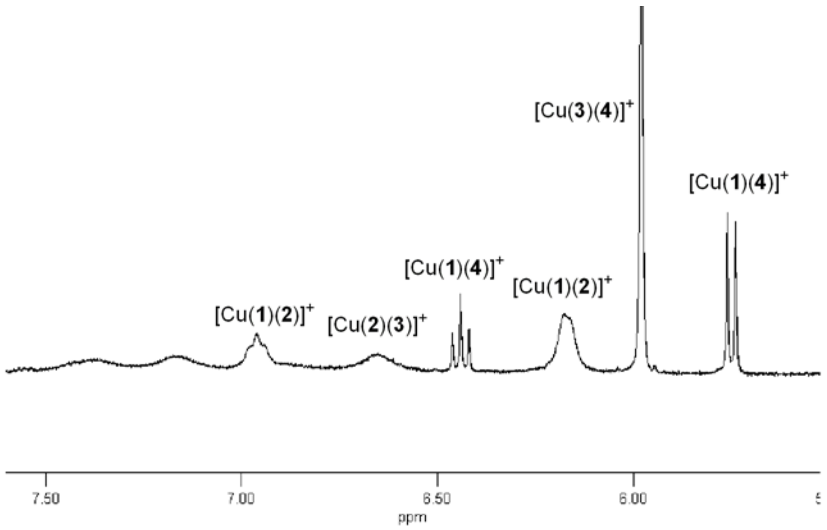

Figure S2: Partial ^{1}H NMR spectrum (CD_3CN, 298 K) of an equimolar mixture of **1**, **2** (R = H), **3** and **4** in the presence of two equivalents of Cu^{+}.

Top

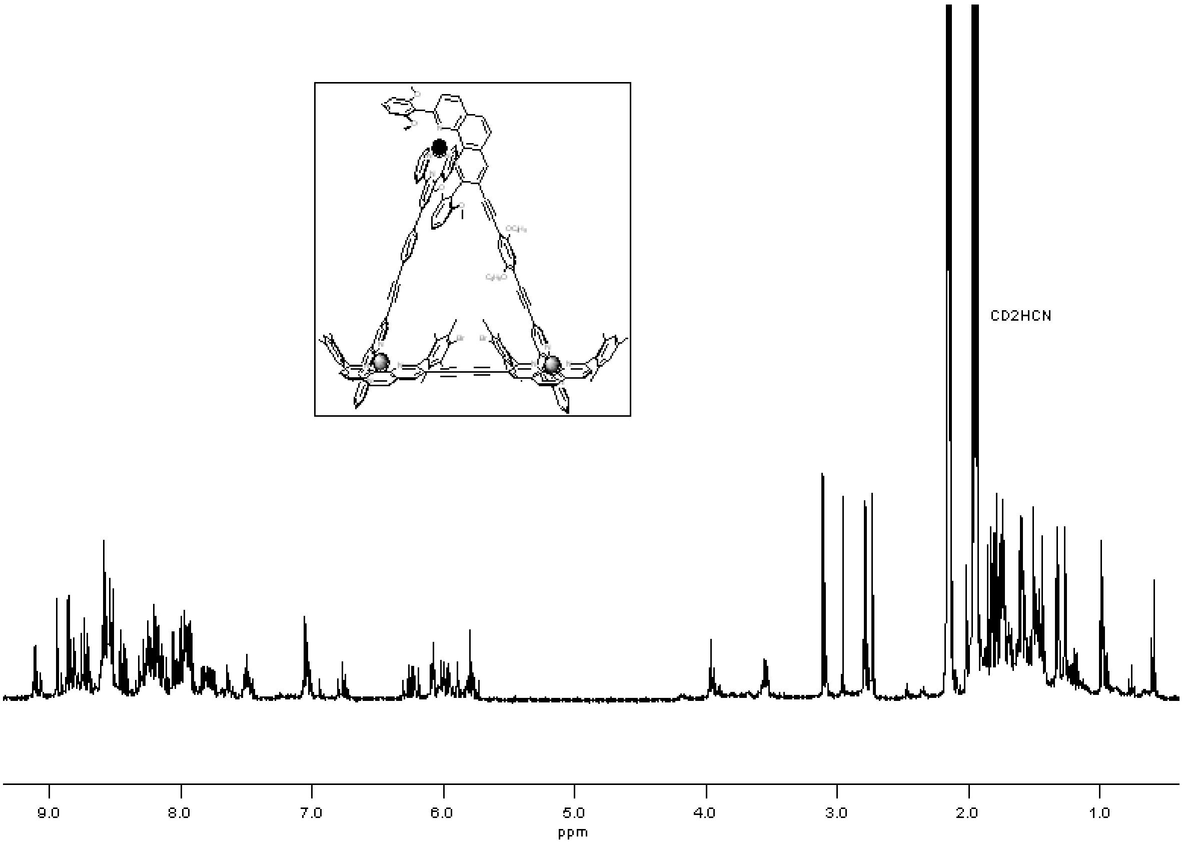

Bottom

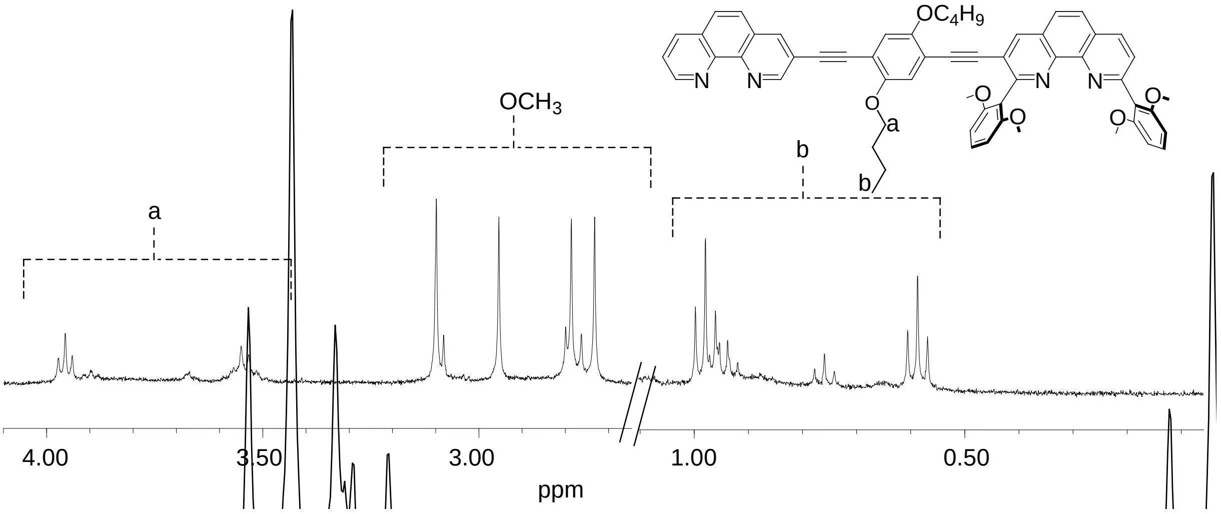

Figure S3: *Top*: ^{1}H NMR spectrum (400 MHz, CD_3CN, 298 K) of scalene triangle **T** = $[Cu_2Zn(\mathbf{5})(\mathbf{6})(\mathbf{7})](OTf)_2(PF_6)_2$ (full); *Bottom*: Partial ^{1}H NMR spectrum of scalene triangle **T**.

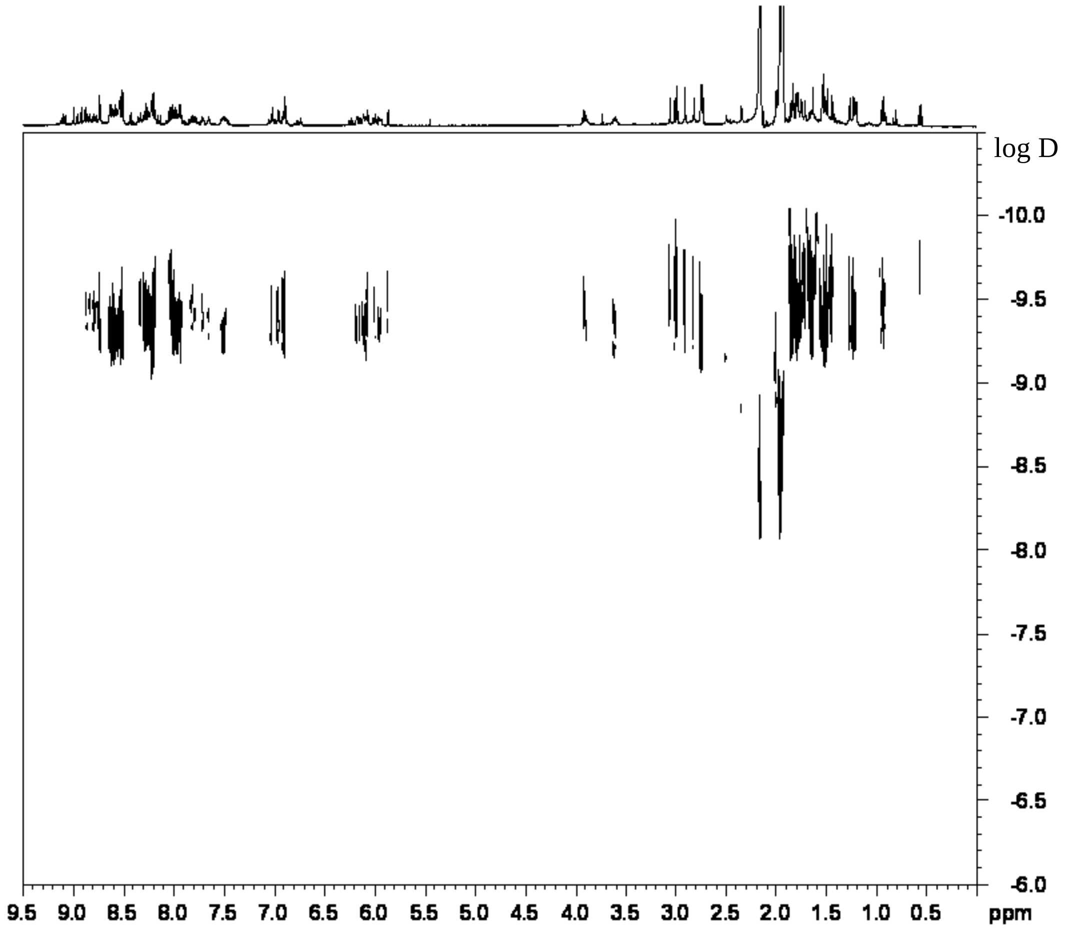

Figure S4: DOSY NMR of triangle **T**.

ESIMS spectra

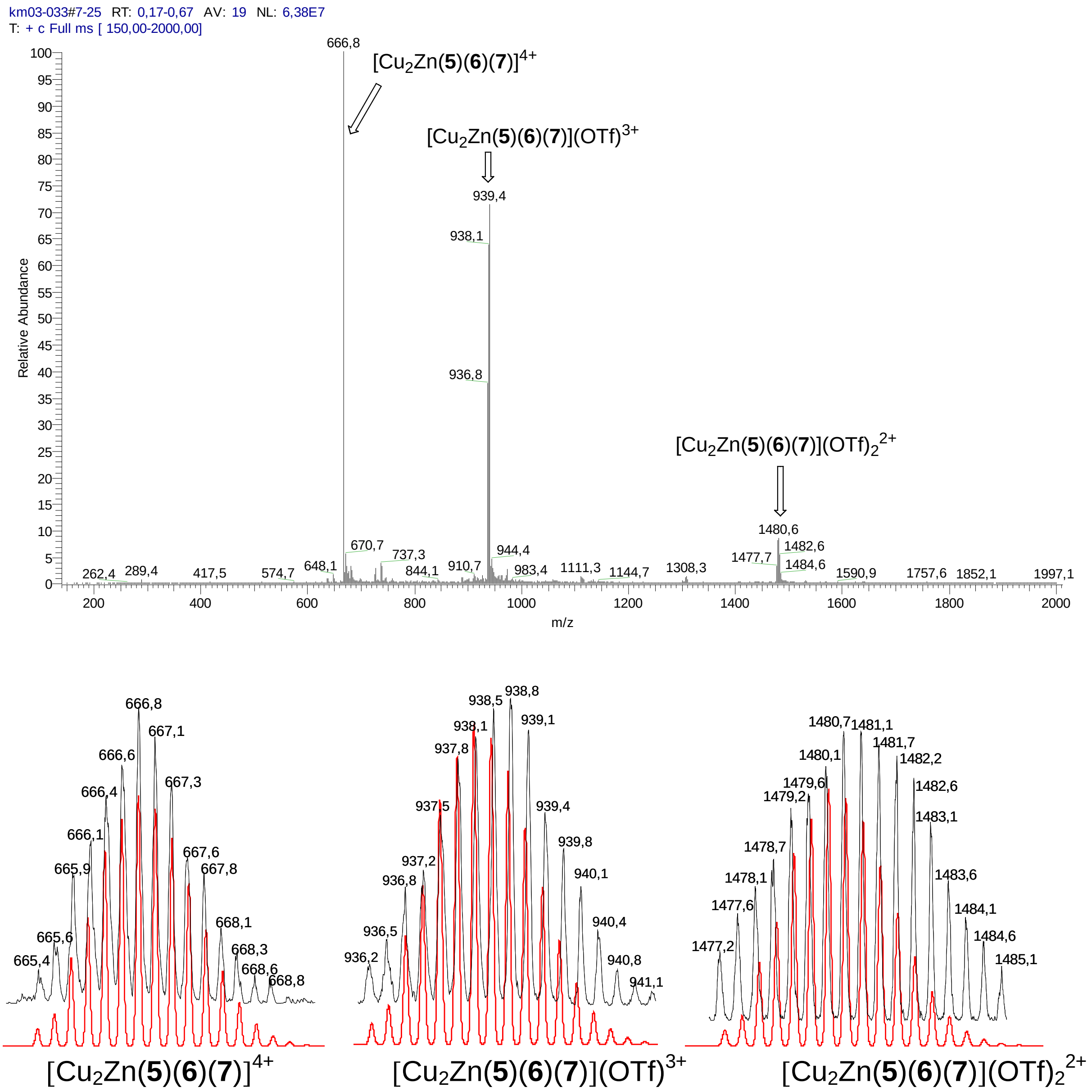

Figure S5: ESIMS spectrum of **T** = [Cu_2Zn(**5**)(**6**)(**7**)]$(OTf)_2(PF_6)_2$ (in acetonitrile) and experimental isotopic distributions (black lines) along with calculated isotopic distributions (red lines) for the different charged species obtained after the loss of counter anion(s).

Differential pulse voltammetry (DPV)

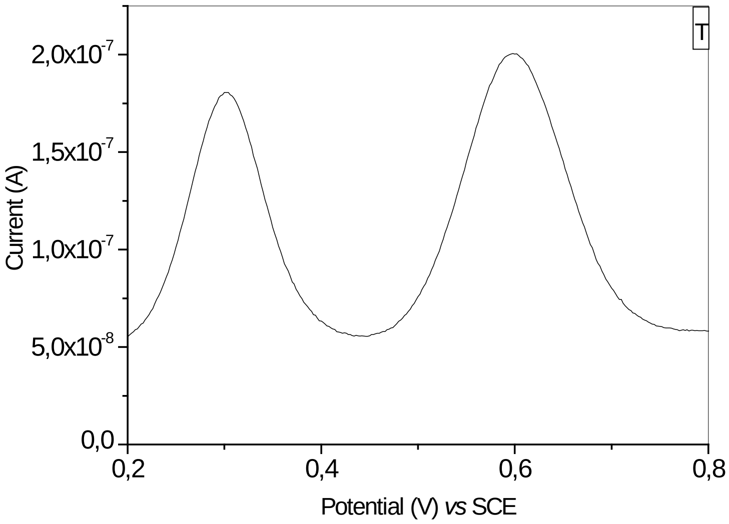

Figure S6: Differential pulse voltammetry (DPV) investigation of scalene triangle **T** = $[Cu_2Zn(\mathbf{5})(\mathbf{6})(\mathbf{7})](OTf)_2(PF_6)_2$. The experiment was carried out in dry acetonitrile with 0.1 M $n$$Bu_4NPF_6$ as electrolyte against a Ag wire as a quasi-reference electrode and 1,1'-dimethyl ferrocene as internal standard (scan rate of 20 mV s^{-1} and a pulse height of 2 mV).

Deconvolution of the DPV

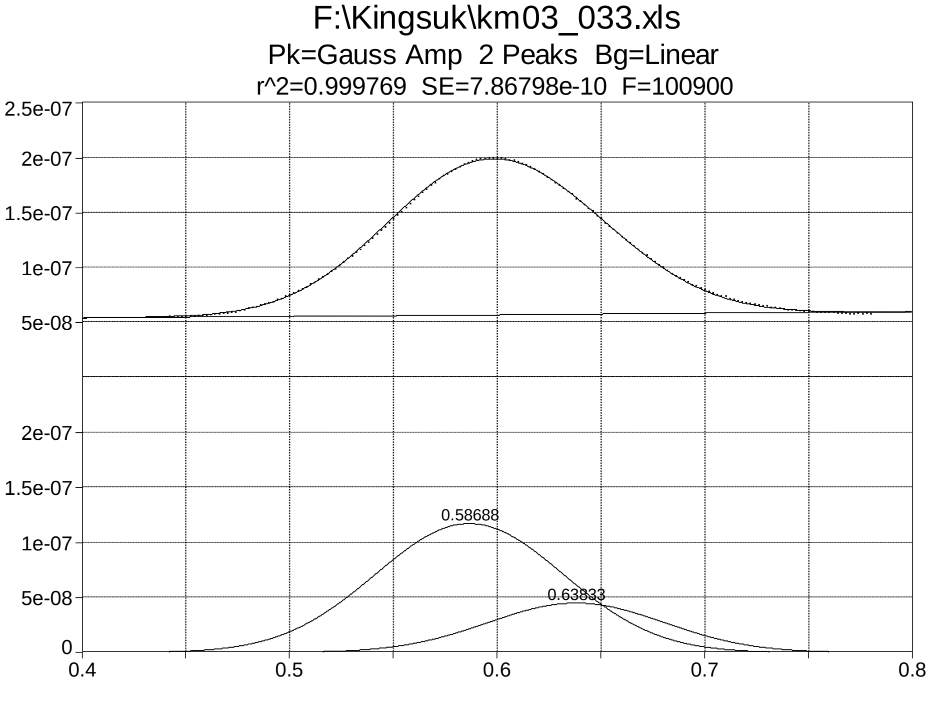

Figure S7: Deconvoluted differential pulse voltammogram of scalene triangle **T** = $[Cu_2Zn(\mathbf{5})(\mathbf{6})(\mathbf{7})](OTf)_2(PF_6)_2$.

Fitted parameters

r^2 Coeff Det	DF Adj r^2	Fit Std Err	F-value
0.99976927	0.99975788	7.868e-10	1.009e+05

Peak	Type	a_0	a_1	a_2
1	Gauss Amp	1.1717e-07	0.58688128	0.04530383
2	Gauss Amp	4.4872e-08	0.63832716	0.04239249
B	Linear Bg	4.831e-08	1.4757e-08	

Measured values

Peak	Type	Amplitude	Centre	FWHM	Asym50	FW Base	Asym10
1	Gauss Amp	1.1717e-07	0.58688128	0.10668236	0.99999994	0.21354696	0.99999997
2	Gauss Amp	4.4872e-08	0.63832716	0.09982669	1.00000001	0.19982391	1.00000004

Peak	Type	Analytic Area	% Area	Int Area	% Area	Centroid	Moment2
1	Gauss Amp	1.3306e-08	73.6186746	1.3298e-08	73.6150632	0.58697359	0.00203817
2	Gauss Amp	4.7682e-09	26.3813254	4.7662e-09	26.3849368	0.63826390	0.00203817
	Total	1.8074e-08	100.000000	1.8064e-08	100.000000		

Parameter statistics

Peak 1	Gauss Amp				
Parm	Value	Std Error	t-value	95	
Amp	1.1717e-07	2.0526e-08	5.70835679	7.664e-08	1.577e-07
Ctr	0.58688128	0.00651154	90.1293794	0.57402343	0.59973913
Wid	0.04530383	0.00169311	26.7576850	0.04196056	0.04864709

Peak 1	Gauss Amp				
Parm	Value	Std Error	t-value	95	
Amp	4.4872e-08	2.3787e-08	1.88645478	−2.097e-09	9.1842e-08
Ctr	0.63832716	0.01159186	55.0668608	0.61543760	0.66121673
Wid	0.04239249	0.00260495	16.2738250	0.03724869	0.04753629

Baseline	Linear Bg				
Parm	Value	Std Error	t-value	95	
a0	4.831e-08	7.8533e-10	61.5152436	4.6759e-08	4.986e-08
a1	1.4757e-08	1.2183e-09	12.1130695	1.2352e-08	1.7163e-08

Analysis of variance

r^2 Coeff Det	DF Adj r^2	Fit Std Err
0.99976927	0.99975788	7.868e-10

Source	Sum of Squares	DF	Mean Square	F
Regr	4.37238e-13	7	6.2462571e-14	100900.41
Error	1.0090543e-16	163	6.1905171e-19	
Total	4.373389e-13	170		

Details of fit

Set Convergence	State	Iterations	Minimization	Extent
1E-6	Stopped	500	Least Squares	1/1

Curvature Matrix	Constraints		Violated
Sparse-Roots	25.0000-5.00000-50.0000	- None - None	0

Computational results

To get some insight into the geometry of the triangle we performed force-field computations (MM+ as implemented in Hyperchem 7.52®, Hypercube, Inc.) and molecular dynamics simulations. For the computations, the long alkyl chains of ligand **6** were replaced by methyl groups and no symmetry constraints were set.

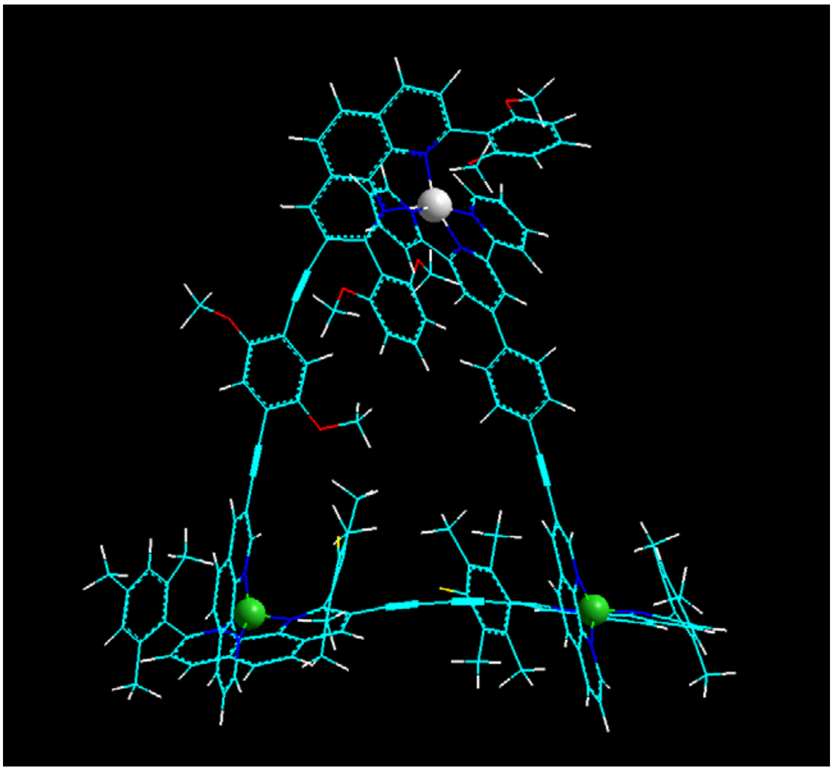

Figure S8: Representations of the energy-minimized structure of the scalene triangle **T** (*syn*); copper(I) ions – green, zinc(II) ion – white.
